# Bioaccumulation Pattern and Health Risk Assessment of Heavy Metals in *Cirrhinus mrigala* at Panjnad Headworks, Bahawalpur, Pakistan

**DOI:** 10.3390/toxics11070596

**Published:** 2023-07-07

**Authors:** Saima Naz, Ahmad Manan Mustafa Chatha, Durali Danabas, Muhammad Farhan Khan, Youhou Xu, Peng Zhu, Laiba Shafique

**Affiliations:** 1 Guangxi Key Laboratory of Beibu Gulf Marine Biodiversity Conservation, Beibu Gulf University, Qinzhou 535011, China; saima.naz@gscwu.edu.pk (S.N.); xuxuyoutiao@163.com (Y.X.); 2 Department of Zoology, Government Sadiq College Women University, Bahawalpur 36100, Pakistan; 3 Department of Entomology, Faculty of Agriculture and Environment, The Islamia University of Bahawalpur, Bahawalpur 63100, Pakistan; manan.chatha@iub.edu.pk; 4 Fisheries Faculty, Munzur University, TR62000 Tunceli, Turkey; dalid07@gmail.com; 5 Department of Chemistry, Gomal University, Dera Ismail Khan 29050, Pakistan; farhankhanbgu@gmail.com

**Keywords:** trace elements, accumulation, freshwater, fish, aquatic toxicity

## Abstract

Heavy metal accumulation in freshwater ecosystem has become one of the major aquatic environmental concerns for freshwater flora and fauna due to their higher stability and bioaccumulation as well as bio-magnification properties. Furthermore, passing through the food web, these heavy metals affect human populations ultimately. This study assessed the heavy metal accumulation in *Cirrhinus mrigala* in spring, autumn, and winter at different locations (I, II, and III) of Panjnad headwork. Furthermore, the human health risk assessment for the consumption of *C. mrigala* from the sampling locations was also carried out. Fish were collected from upper (I), middle (II), and lower (III) stream of Panjnad on a monthly basis. The current study evaluated the accumulation of Aluminum (Al), Arsenic (As), Barium (Ba), and Lead (Pb) in various fish organs (liver, kidney, gills, fins, skin, muscles and bones) and assessed their potential hazard to human health through health risk assessment indicators. The results demonstrated a significant difference (*p* < 0.05) in heavy metal accumulation in different fish organs, seasons, and locations. The accumulation of Al, As, Ba, and Pb were considerably higher in liver and kidney as compared to the other body organs and followed a trend of liver > kidney > gills > fins > skin > bones > muscle and the overall mean concentrations of metals in different body tissues of *C*. *mrigala* were in the order of Al > As > Ba > Pb. The results also concluded that *C. mrigala* caught from the Panjnad headwork is not safe for human consumption due to higher values of *TTHQ_Ing_* (3.76), *THQ_Ing_* for Ba (3.27) and *CR_Ing_* for As (6.4742).

## 1. Introduction

Rivers are a crucial global source of fresh water, but in modern times, the presence of heavy metal contamination in river ecosystems has emerged as a significant challenge for humankind. Due to their toxic and non-biodegradable nature, heavy metals pose a serious threat to both plant and animal life, even at very low concentrations. The sources of heavy metals in rivers can be either natural or anthropogenic in nature, with untreated industrial effluent discharges being a significant contributor to heavy metal contamination. This can lead to serious health problems as these toxic metals can enter the bodies of humans and animals through the food chain [1]. The significant presence of trace elements in riverine waters is a critical concern for environmental pollution and poses a risk to the health of animals and humans alike. For example, in the Panjnad River of Pakistan, concentrations of Al, As, and Pb were examined to be significantly higher than the upper limits for safe human consumption set by World Health Organization and US EPA [2,3]. In order to study the impact of human activities on this significant river of Pakistan, it is imperative to implement effective measures for prevention [4]. Metals can generally be categorized as either biologically essential or nonessential. Nonessential metals such as aluminum (Al), cadmium (Cd), mercury (Hg), tin (Sn), and lead (Pb) do not have any known specific biological functions and their toxicity increases as their concentrations rise. On other hand, essential metals including chromium (Cr), zinc (Zn), nickel (Ni), copper (Cu), cobalt (Co), and iron (Fe) have established biological roles, and their toxic effects arise in response to either insufficient or excessive concentrations [5] which could be more severe than non-essential heay metals. Lead is present in the environment through both natural and human-induced sources. Exposure to Pb can occur through various pathways such as ingestion of contaminated drinking water, consumption of contaminated food, inhalation of air and dust, and exposure to soil contaminated with Pb from old paint. As one of the most commonly recycled non-ferrous metals, Pb’s secondary production has steadily increased. However, high levels of Pb exposure can lead to toxic effects [6]. The toxicity of Pb can cause defects in crucial organs of fish, resulting in abnormalities such as irregular and abnormal fins, heads, tails, and various spinal issues [7]. Arsenic (As) is a highly toxic heavy metal that significantly pollutes aquatic environments, making it one of the most prominent toxicants among heavy metals [8,9]. According to the World Health Organization (WHO), As is classified as one of the most hazardous chemicals to public health [9]. Arsenic is a toxic metalloid that is extensively present in various bodies of water, such as rivers, canals, ponds, groundwater, lakes, and seawater. This is primarily due to the unregulated discharge of industrial wastes and pesticides into aquatic environments [10]. As a heavy metal contaminant, As has significant adverse impacts on fish, affecting their morphology, behavior, growth, histopathology, and gene expression levels [11]. The review of literature highlighted a lack of scientific data on the overall impact of As toxicity on commercially important and popular fish species. Arsenic and Pb are the most prevalent pollutants present in various freshwater sources across Pakistan. In addition to As and Pb, industrial and river water samples collected from Punjab, Pakistan, were found to contain toxic levels of Al and barium (Ba). Aluminum is a detrimental metal to aquatic ecosystems, known to cause toxicity events with severe ecological implications [12]. Exposure to Al has been found to result in various physiological changes in different fish species. These alterations can affect the cardiovascular, hematologic, respiratory, ion-regulatory, reproductive, metabolic, endocrine, and gill systems [13]. There is limited research on Al concentrations in the edible tissues of fish in Pakistan. Barium was found abundantly in various samples of bed sediment, water, and plankton from Panjnad. The concentration of Ba in aquatic organisms is based on their age, tissue type, and external environmental conditions [14]. Research on the behavior of Ba in freshwater ecosystems has been limited. A study examining Ba levels in both water and fish, as part of a more comprehensive investigation of Ba in an aquatic-terrestrial ecosystem, suggested that the concentration of Ba increased from the water to the fish [15]. The concentration of Ba in the muscle tissue of juvenile specimens was 2.5 times higher than that of adult individuals, indicating a significant difference [16]. The ability of Ba compounds to easily dissolve in water can potentially result in detrimental health impacts on individuals [17].

Fish have been widely used as biological indicators to determine the presence of high levels of heavy metals in the aquatic environment [18]. Due to their varying sizes, ages, and positions in the food chain, different species of fish have been utilized to study the correlation between metal concentrations in water and fish, both in laboratory and field study [3,4,19]. Furthermore, the acidic nature of the aquatic environment may also lead to the absorption of free divalent ions of various heavy metals by fish gills [20,21].

In countries such as Pakistan, fish, shellfish, and other aquatic organisms constitute a significant portion of the daily diet of the population. The consumption of heavy-metal-contaminated fish over an extended period of time can result in the accumulation of heavy metals in humans. Despite being a subsector of agriculture and contributing only 1% to the country’s GDP, the fisheries sector in Pakistan is rich in marine and freshwater resources. In 2020, the total fish production in Pakistan was estimated at 701,726 metric tons, with 474,025 metric tons being derived from marine fisheries and the rest from inland freshwater sources (Pakistan Economic Survey, 2019–20) [22]. Research showed that fish are a common dietary component, so it is not surprising that contaminated fish could serve as a hazardous source of certain toxic heavy metals in our diet [23]. Heavy metals tend to accumulate in both the soft and hard tissues of fish through the process of bioaccumulation. The build-up of heavy metals in fish serves as a means of detecting the concentration of these metals in aquatic environments. Furthermore, these metals can be passed on from fish to their predators within the food chain [24]. *Cirrhinus mrigala* is a freshwater fish species that holds significant economic value in numerous countries, including Pakistan [18]. Mrigals are considered detritivore fish that typically inhabit the bottom of aquatic environments and are well-suited for composite aquaculture practices [25]. *Cirrhinus mrigala* exhibits more capacity to tolerate a broad range of experimental conditions. Additionally, this fish species holds significant commercial value due to its ease of cultivation and is well-regarded for its flavor [26].

Various studies have been conducted to evaluate and analyze heavy metal concentrations in different rivers of Punjab, Pakistan. However, limited information was available on the presence of toxic metals, such as As, Pb, Al, and Ba, in *C. mrigala* living in the Panjnad River. Located at the far end of the Bahawalpur district in Punjab, the Panjnad River is formed by the successive merging of five rivers. Due to the significant discharge of numerous metallic compounds into the rivers of Pakistan, the freshwater ecosystem has been severely affected. The accumulation and toxicity of heavy metals in aquatic ecosystems pose a significant concern. Consequently, this study aims to contribute to our understanding of the health risks associated with heavy metal (As, Ba, Al, and Pb) contamination in *C. mrigala* within its environment as little information is available on the toxic profile of these heavy metals at the study sites.

## 2. Materials and Methods

### 2.1. Study Area

**Figure 1:**
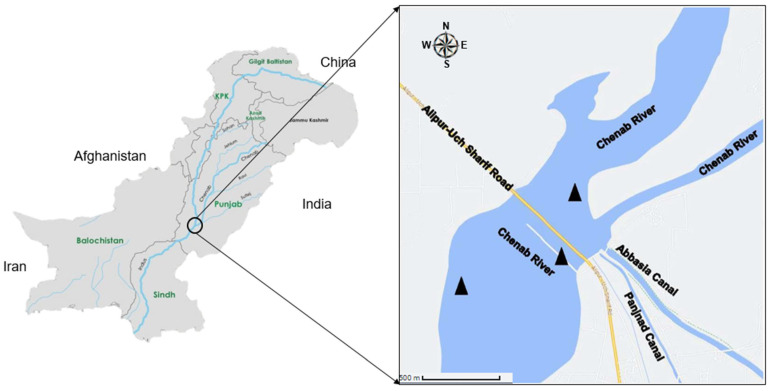
Map of the study area (Panjnad headwork). The sampling sites are represented by.

The Panjnad headwork is situated at an altitude of 99 m above sea level. The area experiences an average annual rainfall of 3.56 mm, an average annual temperature of 29.49 °C, and an average annual relative humidity of 27.64%. As it is the confluence point of various rivers, the Panjnad headwork receives a diverse range of pollutants from these rivers. The primary sources of pollution in the Panjnad headwork are agricultural, domestic, and industrial wastes from various cities, such as Gujarat, Faisalabad, Jhang, and Multan. These wastes come from industries such as textile, dyeing, petrochemical, hosiery, oil refineries, sugar and flour mills, distilleries, tannery, rubber, and plastics. They are discharged into the river ecosystem, which further contributes to the pollution in the region [27]. For this study, samples were collected from three stations (SI, SII, and SIII) between September 2021 and May 2022 (Figure 1). Throughout the study period, the sampling stations were visited every month. The selection of sites was based on different levels of pollutant accumulation. The current study conducted three replications during each season at each site, resulting in a total of nine replications for each experimental factor.

### 2.2. Collection and Digestion of Samples

To conduct the study, we chose *C. mrigala* being the most common and abundant fish species at the study site. This species was easily caught and is commonly consumed in the surrounding areas. Fish samples were collected randomly from three different locations of Panjnad river in three different seasons (spring, autumn, and winter). The sampling of fish was carried out using a gauze net with the dimension of 100 m × 6 m, having a mesh-size of 60 mm. Samples of *C. mrigala* were collected randomly from each of the three sampling sites three times a month from September 2021 to May 2022. The samples were then placed in an icebox (Coleman Ice Box 48 Quart) with ice cubes placed at the bottom of the box to preserve the freshness of collected samples. The collected samples were then transported to the zoology laboratory at department of zoology, Government Sadiq College Women University, Bahawalpur and kept in a deep freezer (Haier −25 °C Biomedical Freezer) at a temperature of −20 °C for digestion and further analysis. In the laboratory, the species of the sampled fish were identified using a fish identification manual [28]. The liver, kidney, gills, fins, skin, muscles, and bones of the fish were dissected for analysis of heavy metal content. The acid digestion method was used to digest the dissected organs with analytical-grade HClO_4_ (60%, Daejung, Siheung-si, Republic of Korea) and HNO_3_ (65%, Sigma-Aldrich, St. Louis, MO, USA) in a ratio of 1:3 *v/v*. This method was performed for 5 ± 0.25 h. The digested sample was then heated on a hot plate (ANEX Deluxe Hot Plate AG-2166-EX) at 200 °C for 30 ± 5 min. Once completely digested, the samples were cooled down to 25 ± 2 °C. The cooled samples were then filtered through Whatman No. 42 filter paper. As the atomic absorption spectrophotometer (AAS) requires diluted samples for the analysis, the samples were diluted with double distilled water up to 50 mL for further analysis. After dilution, the digested samples were sealed in analytical-grade 50 mL glass vials and sent to the Central Laboratory, Mian Nawaz Sharif University of Agriculture (MNSUA), Multan, Punjab, Pakistan for heavy metal detection.

### 2.3. Heavy Metal Detection in Fish Organs

The concentration of heavy metals (Al, As, Ba, and Pb) in different organs of *C. mrigala* was detected at Central Laboratory, MNSUA, Multan, using an acetylene air flame AAS (Analytik Jena: NovAA 400 P) according to the recommended instrument parameters such as detection limits (Table 1). The precision and accuracy of the AAS analysis was confirmed by comparing the results with reference material (CRM IAEA 407) guided by the International Atomic Agency (IAEA). The heavy metal determination showed an acceptable performance, as the analytical results of the blanks and standards for the studied metals were within the certified values range of 95–101% recovery.

### 2.4. Health Risk Assessment

Various health risk assessment indicators were applied to assess heavy metal exposure, carcinogenic effects, and non-carcinogenic effects of studied heavy metals in fish muscle.

### 2.5. Estimation of Oral Ingestion Exposure

Fish is widely consumed in Pakistan, especially in areas which are close to the rivers and other freshwater resources. Fish is among the cheapest and highest-quality sources of protein, vitamins, and other dietary minerals (Roos et al., 2007).

In order to estimate the heavy metal ingestion exposure to the fish, the following equations were used.

(1) $${Ing}_{ex}=\frac{Con.\times IR\times EF\times ED}{BW\times AT}$$

where *Ing_ex_* is the ingestion exposures of each heavy metal via oral intake of fish. *Con.* represents the concentration (mg/kg) of respective heavy metals in fish muscles. Various formula constants were also applied in the equation for the estimation of ingestion exposure of heavy metals in fish muscles (Table 2).

### 2.6. Target Hazardous Quotients via Ingestion (THQ_Ing_) 

The *THQ_Ing_* for the oral ingestion exposure of the heavy metals was estimated using Equation (2). The equation is based on USEPA Region III risk-based concentration criteria assuming the duration of heavy metal exposure of fish consumers is 30 years.

(2) $${THQ}_{Ing}=\frac{{Ing}_{ex}}{Rf_{o}D}$$

where *Ing_ex_* is the estimated ingestion exposures through oral intake and is derived from Equation (1). Reference dose (*Rf_o_D)* is the permissible limit (mg) of each metal per kg per day of fish. For the metals in the present study, *Rf_o_D* (mg/kg/day) are; Al = 1.0, As = 0.0003, Ba = 0.2, and Pb = 0.0035 [29]. A *THQ_Ing_* < 1 shows non-lethal health impact for human consumption, while a *THQ_ing_* > 1 represents a significant impact of contaminated fish on the exposed consumers [29].

### 2.7. Total Target Hazardous Quotients (TTHQ_Ing_)

The *TTHQ_Ing_* is estimated as a summation (∑) of *THQ_Ing_* of studied heavy fish muscles. The *TTHQ_Ing_* were estimated based on the following equations.

(3) $$T{THQ}_{Ing}=\sum_{i}^{k}{THQ}_{Ing}$$

where “*k*” represents the total number of heavy metals in this study, and it is the sum of each metal *THQ_Ing_* for each sampling location. A *TTHQ_Ing_* < 1 shows non-lethal health impact for human consumption, while a *TTHQ_Ing_* > 1 represents a significant impact of contaminated fish on the exposed consumers [29].

### 2.8. Carcinogenic Risk (CR)

The CR represents the potential risk posed by heavy metal intake to cause cancer. The CR was calculated based on Equation (4) for fish consumers [3,30].

(4) $${CR}_{Ing}={Ing}_{ex}\times {SF}_{C}$$

where *SF_C_* is the cancer risk slope factor (0.0085 and 1.5 (mg/kg per day) for Pb and As, respectively) [31,32,33]. For Al and Ba, *SF_C_* is not known as these heavy metals are dietary requirements, and the cancer risk slope is not defined for these heavy metals. 

### 2.9. Statistical Analysis

The assessment of heavy metal contamination in different organs of *C. mrigala* at different locations (SI, SII, and SIII) and different seasons (spring, autumn, and winter) was based on the analysis of individual heavy metal concentration in the fish organs. The heavy metals concentration in the liver, kidney, gills, fins, skin, muscles, and bones of *C. mrigala* were compared using a one-way analysis of variance (ANOVA) and the Duncan’s new multiple range test with *α =* 0.05 was performed to compare heavy metal accumulation in different fish organs in all sampling sites and seasons using IBM SPSS (Version 25.0). Furthermore, correlation analysis was also conducted to observe the relation between heavy metal concentration in different organs of *C. mrigala* in various sampling months (October 2021–May 2022) using Minitab (version 19.11).

## 3. Results

A study was conducted to investigate the aquatic pollution at Panjnad headwork, Punjab, Pakistan, caused by four (Al, Ba, As, and Pb) heavy metals in different organs (liver, kidney, gills, fins, skin, muscles, and bones) of *C. mrigala* during autumn, winter, and spring seasons. The investigation of Al contamination in different organs of *C. mrigala* at three different locations (SI, SII, and SIII) showed that highest concentration of Al (µg·g^−1^) at SI was found in fish kidney with the overall mean (151.90 ± 6.10), followed by liver, gills, skin, fins, and bones, and the lowest concentration was found in fish muscles (72.38 ± 6.11). Similar pattern of Al concentration was also recorded in SII and SIII, with a slight change in Al concentration in SII where the lowest Al concentration was recorded in fish bones (72.25 ± 6.21). During comparison of Al concentration at different locations in different seasons, the highest Al concentration in fish kidney at SI was found in spring season (156.51 ± 6.02) followed by winter, and the lowest concentration was found in fish muscles in autumn (66.84 ± 4.94). Similar results were also recorded for other two locations (SII and SIII) with a slight difference in the lowest Al concentration at SII, which was recorded in fish bones (66.62 ± 6.30) instead of muscles. Among the comparison of different locations (SI, SII, and SIII), the highest Al concentration was found in kidney at SIII (152.31 ± 4.89) while the lowest concentration was recorded in fish bones (72.25 ± 6.21) at the SII location (Table 3). 

The investigation of As contamination in different organs of *C. mrigala* at three different locations (SI, SII, and SIII) showed that the highest concentration of As (µgg^−1^) at SI was found in fish kidney with the overall mean (26.86 ± 5.36), followed by liver, gills, fins, skin, and bones, and the lowest concentration was found in fish muscles (13.77 ± 3.53). Similar patterns of As concentration were also recorded in SII and SIII, with the highest concentrations in kidney (26.70 ± 4.60 and 26.73 ± 4.87) and the lowest in fish muscles (13.25 ± 2.83 and 13.63 ± 3.77) at SII and SIII, respectively. During comparison of As concentration at different locations in different seasons, the highest As concentration in fish kidney at SI was found in spring season (32.71 ± 2.43) followed by winter, and the lowest concentration was found in fish bones in autumn (10.04 ± 1.70). Similar results were also recorded in other two locations (SII and SIII) with the highest concentrations in kidney (31.87 ± 1.81 and 32.08 ± 1.42) and the lowest in fish bones (9.59 ± 2.40 and 9.24 ± 2.52) at SII and SIII, respectively. The comparison of different locations (SI, SII, and SIII) revealed that the highest As concentration was found in kidney at SI (26.86 ± 5.36) while the lowest concentration was recorded in fish bones (9.24 ± 2.52) at the SII location (Table 4). 

The investigation of Ba contamination in different organs of *C. mrigala* at three different locations (SI, SII, and SIII) showed that highest concentration of Al (µgg^−1^) at SI was found in fish liver with the overall mean (5.12 ± 1.88), followed by kidney, gills, fins, skin, and muscles, and the lowest concentration was found in fish bones (3.32 ± 2.01). A similar pattern of Ba concentration was also recorded in SII and SIII, with a slight change in Ba concentration in SII where the lowest Al concentration was recorded in fish muscles (2.70 ± 1.10). During comparison of Ba concentration at different locations in different seasons, the highest Ba concentration at SI was found in the spring season (7.43 ± 0.70) in fish liver followed by winter, and the lowest concentration was found in autumn (1.38 ± 0.83) in fish bones. Similar results were also recorded in other two locations (SII and SIII) with a slight difference in the lowest Ba concentration at SIII, which was recorded in fish muscles (1.19 ± 0.31) instead of muscles. The comparison of different locations (SI, SII, and SIII) revealed that the highest Ba concentration was found in liver at SI (5.12 ± 1.88) while the lowest concentration was recorded in fish muscles (2.70 ± 1.10) at the SII location (Table 5).

The investigation of Pb contamination in different organs of *C. mrigala* at three different locations (SI, SII, and SIII) showed that highest concentration of Pb (µg·g^−1^) at SI was found in fish liver with the overall mean (4.73 ± 1.96), followed by kidney, gills, fins, skin, and muscles, and the lowest concentration was found in fish bones (2.53 ± 1.33). A similar pattern of Pb concentration was also recorded in SII and SIII, with the highest concentration in fish liver (4.34 ± 1.69 and 4.67 ± 1.84) and the lowest concentration in fish bones (2.43 ± 1.42 and 2.63 ± 1.62) for SII and SIII, respectively. During comparison of Pb concentration at different locations in different seasons, the highest Pb concentration at SI was found in fish kidney during spring (7.57 ± 0.42) followed by winter, and the lowest concentration was found in fish muscles in autumn (1.22 ± 0.58). However, SII and SIII locations showed different varying results. During sampling at SII, the highest Pb concentration was found in fish kidney during spring (6.45 ± 0.95) followed by winter, and the lowest concentration was found in fish bones in autumn (0.77 ± 0.32), while at SIII, the highest Pb concentration was recorded in fish liver during spring (6.67 ± 1.84) followed by winter, and the lowest concentration was found in fish bones in autumn (0.80 ± 0.59). Among the comparison of different locations (SI, SII, and SIII), the highest Al concentration was found in liver at SI (4.73 ± 1.96) while the lowest concentration was recorded in fish bones (0.77 ± 0.32) at the SII location (Table 6). Overall, these findings showed that fish liver and kidney are most affected organs for heavy metal contamination in any season or location while fish bones and fish muscles are the least affected fish organs. The results also showed that heavy metal accumulation is significantly higher (*p* < 0.05) in spring while being significantly lower (*p* < 0.05) during autumn.

The relation between sampling months (September–May) and heavy metal (Al, As, Ba, and Pb) accumulation in different organs (liver, kidney, gills, finds, skin, muscles and bones) of *C. mrigala* was analyzed with Pearson correlation. The obtained results showed a combination of significant and non-significant positive as well as negative correlation between sampling months and accumulation of different heavy metals. Overall, most of the significant correlation between sampling month and heavy metal accumulation was observed in spring months as compared to autumn and winter. 

The correlation between sampling months and Al accumulation in fish showed significant negative as well as positive correlation among various months. The highest significant correlation was observed between January and October (*r* = 0.819, *p* = 0.007) in fish liver while the least significant correlation was found between January and April (*r* = 0.674, *p* = 0.047) in fish muscles. It was also observed that most of the significant correlations for Al concentration in various fish organs occurred from January to May (Table 7). 

The correlation between sampling months and As accumulation in fish showed significant negative as well as positive correlation among various months. It was found that January and November had the highest significant correlation for As accumulation (*r* = 0.907, *p* < 0.000) in fish muscles while the least significant correlation was observed between October and May (*r* = 0.672, *p* = 0.047) in fish liver. Like Al, most of the significant correlations for As concentration in various fish organs occurred in later months. (Table 8).

The correlation between sampling months and Ba accumulation in fish showed a number of significant negative as well as positive correlation among various months. For Ba, February and March showed the highest significant correlation for Ba concentration (*r* = 0.984, *p* < 0.000) in skin, while the lowest significant correlation was observed between February and October (*r* = 0.670, *p* = 0.048) in fish liver. Like Al and Ba, most of the significant correlations for Ba accumulation in various fish organs were observed in late winter or in spring. (Table 9).

The correlation between sampling months and Pb concentration in fish organs showed a number of significant negative as well as positive correlations among various months. For Pb, the highest significant correlation was observed in fish kidney between February and March (*r* = 0.956, *p* < 0.000), while the least significant correlation was found between January and March (*r* = 0.672, *p* = 0.047) in fish muscles. Like other heavy metals, it was also observed that most of the significant correlations for Pb concentration in various fish organs occurred from January to March. (Table 10). 

The correlation analysis also showed that most of the significant correlations between sampling months for various heavy metals were found in Ba followed by Pb and As, and the least significant correlation was observed for Al accumulation. 

Fish are among the most consumed and important diets worldwide. The accumulation of heavy metals in fish muscles could pose a serious health risk to the consumers if the heavy metal contamination in fish muscles is above permissible limits. The health risk assessment of studied fish was carried out to evaluate the potential health hazards due to oral exposure of studied heavy metals. The observed oral exposure (*Ing_ex_*) was calculated in milligrams per kilogram per day (mg kg^−1^/day) for ingestion of *C. mrigala*. The results showed that the highest oral exposure through fish consumption was recorded for Al (23.05) followed by As, Ba, and Pb with *Ing_ex_* values of (4.3, 0.98, and 0.89), respectively. However, the highest ***THQ_Ing_*** was found in Ba followed by Pb, Al, and As with the values of (0.44, 0.02, and 0.02), respectively. Furthermore, the ***TTHQ_Ing_*** of the studied heavy metals was recorded to be 3.76. The ***CR_Ing_*** values for Ba and Al were not calculated as these metals and dietary requirement do not have a cancer slope factor. However, the ***CR_Ing_*** of As and Pb was found to be 6.4742 and 0.0076, respectively (Table 11).

The obtained results showed that the *Ing_ex_* values of studied heavy metal were within the permissible limits according to the guidelines of ATSDR (2007) and EFSA (2008) except for Al and As which were higher than the permissible limits of 1 mg·kg^−1^ bw/week and 2.14 μg·kg^−1^ bw /day for Al and As, respectively. *THQ_Ing_* values of Al, As, and Pb showed that these heavy metals are not hazardous. However, Ba was found to be hazardous as its *THQ_Ing_* (3.27 mg·kg^−1^/day) was greater than 1. Similarly, based on higher *THQ_Ing_* value of Ba in the fish muscle samples, the *TTHQ_Ing_* also showed potential hazard for the consumption of the *C. mrigala* from the sampling sites. *CR_Ing_* for As was found to be potentially carcinogenic in *C. mirgala* for human consumption. Overall, the results indicated that the studied sites possess potentially elevated accumulation of heavy metals and that the *C. mrigala* species captured from these sites are not safe for long term human consumption.

## 4. Discussion

Heavy metal accumulation in freshwater ecosystems has become one of the major aquatic environmental concerns for freshwater organisms such as fish due to their higher stability and bioaccumulation as well as bio-magnification properties [34,35]. The unplanned expansion of urban areas and the rapid growth of industries near the Panjnad headwork on the Chenab River in Bahawalpur have led to significant pollution in the surrounding environment. This pollution is caused by the release of large amounts of untreated hazardous waste and domestic sewage, resulting in detrimental effects on the local ecosystems [36,37]. Current study evaluated the bioaccumulation of heavy metals in different organs of *C. mrigala* in various seasons and sampling locations. Furthermore, the health risk assessment of heavy metal accumulation in fish muscles was also explored to assess the impact of fish consumption from the selected locations.

The present study concluded that kidney and liver of *C. mrigala* accumulated significantly more heavy metals as compared to other organs. It is now widely known that the kidney and the liver are more actively involved in metabolic process of fish as compared to other organs such as muscles, bones, and skin [38]. A number of studies on various fish species have concluded that the kidney and the liver usually accumulate higher concentrations of heavy metals as compared to other fish organs [39]. Kidney and liver are the primary site of metal detoxification in fish body, due to which these organs tend to accumulate higher levels of various pollutants including heavy metals [40].

Our primary concern is the accumulation of toxic compounds in fish in their flesh (muscle), which is the most consumed organ. However, in comparison to other tissues, muscles generally contain lower levels of metals [41]. It is possible for muscle levels to be undetectable even when the concentration in the liver is high [42]. In contrast, to other fish organs, muscles are not considered a primary site for metal intake in fish, as they typically contain lower levels of trace elements and metals compared to other organs but in a human health perspective the accumulation of heavy metals in fish muscles becomes more concerned.

The current study showed that the muscles of *C. mrigala* accumulated the lowest quantity of heavy metal pollutants. Similar results have been concluded previously in various fish species, suggesting that fish muscles are among the least affected body organ by heavy metal pollution and have no or fewer active mechanisms for releasing them [43,44]. Li et al. [45] concluded similar results while studying Cd, Pb, As, and Hg accumulation in *Carassius auratus*, *Pelteobagrus fulvidraco,* and *Hippelates nobilis* sampled in the Nansi Lake in China.

The current study recorded higher concentration of heavy metals (Al, As, Ba, and Pb) during spring as compared to the autumn and winter. Higher levels of physical and physiological activities performed by the fish during the hot weathers as compared to the winter and autumn could a plausible explanation for this higher heavy metal accumulation in spring and lower accumulation in autumn season [18,46,47]. These findings are coherent with a number of other studies which have concluded that accumulation of heavy metals is significantly higher in spring as compared to autumn and winter in the Mediterranean fish [48,49]. Similarly, another study has shown that higher growth rate in spring and summer in fish may cause significant more heavy metal accumulation in these seasons as compared to cold seasons [46].

The bio-accumulation of metals in fish bodies is a complex process, and its variation depends on the mode of action of the metals [50]. The toxicity of metals found in the fish species studied showed a direct correlation with the eco-toxicity of the Panjnad, as well as the metabolism, feeding patterns, and ecological requirements of the fish species under investigation. The current study revealed significant differences in metal accumulation among various organs, likely attributable to variations in the physiological functions of these organs within the fish body. Specifically, the liver emerged as the primary target organ for metal toxicity, followed by the kidney, gills, scales, fins, bones, and muscles, in descending order of metal accumulation. Fish muscles and bones exhibited notably lower concentrations of the studied metals. It is important to note that bio-accumulation refers to the uptake of metals by an organism, but within the organism, metals are distributed among various tissues, each with a specific affinity for accumulation [51].

The mean accumulation of various metals in different body organs of *C. mrigala* were in the order of Al > As > Ba > Pb. Naz et al. [3] concluded similar trends for the contamination of these heavy metals in water and sediments at Panjnad. Hence, it can be inferred that the ability of fish to bio-accumulate heavy metals is directly influenced by the concentration of these metals in the water and sediment of the aquatic environment.

The current study is coherent with Mahboob et al. [52] and Chatha et al. [18] who concluded that heavy metal concentration in fish organs was significantly greater in the summer as compared to other seasons. The correlation analysis conducted in this study showed that there is a higher statistically significant correlation between sampling months of late winter (January and February) and spring season (March–May) for accumulation of different heavy metals in various fish organs among sampling months. Fishes such as *C. mrigala* being larger in size tend to accumulate more heavy metals contents due to their widespread column-feeding nature [53,54], which is also observed in the current study, and higher heavy metals accumulation was found in *C. mrigala* as compared to the permissible limits.

In order to assess the impact of heavy metal accumulation in fish to the human health, *Ing_ex_*, *THQ_Ing_*, *TTHQ_Ing_*, and *CR_Ing_* parameters were calculated for the consumption of muscles of *C. mrigala* from Panjnad headwork, Punjab, Pakistan. *THQ_Ing_* readings for Al metal analyzed in *C. mrigala* were the highest for fish consumers, following the arrangement Al > As > Ba > Pb. Similarly, Harmanescu et al. [55] concluded a similar pattern of health assessment of heavy metals such as Pb and Cd. The *THQ_Ing_* analysis showed that Ba values for *THQ_Ing_* were >1, suggesting a potential hazard for the Ba intake. Apart from Ba, the *THQ_Ing_* values for other heavy metals were <1, which suggests that the accumulation of these heavy metals in fish muscles do not pose any potential health hazards to its consumers. The *TTHQ_Ing_* was also >1 due to the additive effect of Ba in the mixture of other heavy metals. It suggests that *C. mrigala* collected from the sampling sites were not safe for human consumption. Our results are coherent with [56], who also found lower concentrations of As in fish samples. On the other hand, studies have concluded that As, even at very low concentrations, disrupts the hormonal activity of endocrine glands [57]. Therefore, a number of health issues in organs such as the gastrointestinal tract, respiratory tract, skin, liver, cardiovascular system, hematopoietic system, and nervous system may arise by the chronic exposure to inorganic As even at lower concentrations [58].

It is observed that human populations may encounter trace metals by consuming water or beverages that are rich in metals, inhaling air contaminated with trace metals, or ingesting other food substances [59]. Additionally, there are various pathways through which trace metals can enter fish ponds, extending beyond the water within the pond and the fish feed [60]. Naz et al. [3] found similar health risk pattern for studied heavy metals in water, plankton, and sediments from Panjnad river.

The carcinogenic risk (*CR_Ing_*) of heavy metals (Al, As, Ba, and Pb) was determined using the consumption levels of *C. mrigala*. The study indicated that heavy metals such as Al, Ba, and Pb did not show carcinogenic risks for human health. However, As levels were above the borderline for safe human consumption suggested by USEPA [61] for oral intake. It could be due to the higher toxic properties of As when taken orally. It has been shown that even very little accumulation of As in water could cause carcinogenic effects in humans when taken orally [62].

## 5. Conclusions

It is widely known that various heavy metals such as Al, As, Ba, and Pb accumulate in aquatic organisms when present in fresh water and may cause serious damage to fish organs. The consumption of these polluted fish causes potential health hazards to its consumers. This study concluded that various heavy metals were accumulated at various concentrations in different fish organs. However, health risk assessment suggests that *C. mrigala* was not safe for human consumption due to its associated carcinogenic and non-carcinogenic potential health risks. An understanding of the adverse effects of heavy metals in freshwater organisms and their permissible concentrations in the aquatic environment would be extremely essential for fish conservation, fisheries development, and safe human consumption.

## Figures and Tables

**Table 1:** Condition of atomic absorption spectrometer used for the detection of heavy metal concentration.

Metals	Wavelength (nm)	Gas	Support
Aluminum (Al)	309.3	Acetylene	Air
Arsenic (As)	193.7	Acetylene	Air
Barium	553.6	Acetylene	Air
Lead (Pb)	217	Acetylene	Air

**Table 2:** Constants used in the formula for the calculation of the health risk assessments.

Term Used	Definition	Value
IR	Ingestion rate	0.02 kg/day
EF	Exposure frequency	365 days/year
ED	Exposure duration	30 years
BW	Body weight	70 kg
AT	Average days	10,950 days

**Table 3:** Al concentration (mg kg^−1^) in tissue samples (Means ± SD) of *C. mrigala* caught from different sites at Panjnad headwork, Pakistan.

Sites	Seasons	Fish Tissues						
		Liver	Kidney	Gills	Fins	Skin	Muscles	Bones
**S1**	Autumn	125.58 ± 3.73 ^bCP^	146.24 ± 2.71 ^aBP^	116.70 ± 5.39 ^cCP^	90.40 ± 4.39 ^eBP^	100.86 ± 2.96 ^dCP^	66.84 ± 4.94 ^fBP^	68.33 ± 5.78 ^fBP^
	Winter	136.82 ± 3.43 ^bBP^	152.95 ± 4.01 ^aAP^	127.78 ± 3.99 ^cBP^	101.86 ± 3.05 ^eAP^	106.80 ± 4.5 ^dBP^	73.13 ± 3.82 ^fAP^	75.26 ± 4.35 ^fAP^
	Spring	141.83 ± 4.92 ^bAP^	156.51 ± 6.02 ^aAP^	132.82 ± 3.48 ^cAP^	104.38 ± 3.45 ^eAP^	111.69 ± 2.89 ^dAP^	77.17 ± 4.6 ^fAP^	77.60 ± 3.02 ^fAP^
	**Mean**	**134.74 ± 7.95 ^X^**	**151.90 ± 6.10 ^X^**	**125.77 ± 8.04 ^X^**	**98.88 ± 7.13 ^X^**	**106.45 ± 5.64 ^X^**	**72.38 ± 6.11 ^X^**	**73.73 ± 5.91 ^X^**
**S2**	Autumn	128.43 ± 1.95 ^bBP^	146.59 ± 3.93 ^aCP^	116.62 ± 6.58 ^cBP^	92.55 ± 4.96 ^eBP^	101.87 ± 3.08 ^dBP^	67.96 ± 4.42 ^fBP^	66.62 ± 6.30 ^fBP^
	Winter	136.76 ± 3.80 ^bAP^	150.21 ± 2.49 ^aBP^	131.45 ± 5.95 ^cAP^	98.94 ± 3.79 ^eAP^	108.82 ± 3.54 ^dAP^	73.15 ± 2.92 ^fAP^	74.35 ± 3.29 ^fAP^
	Spring	137.56 ± 7.49 ^bAP^	155.77 ± 3.34 ^aAP^	126.59 ± 8.69 ^cAQ^	102.13 ± 3.64 ^eAP^	108.75 ± 5.09 ^dAP^	76.95 ± 5.08 ^fAP^	75.77 ± 4.53 ^fAP^
	**Mean**	**134.25 ± 6.37 ^X^**	**150.85 ± 4.99 ^X^**	**124.89 ± 9.33 ^X^**	**97.87 ± 5.70 ^X^**	**106.48 ± 5.08 ^X^**	**72.69 ± 5.53 ^X^**	**72.25 ± 6.21 ^X^**
**S3**	Autumn	126.35 ± 2.67 ^bBP^	147.52 ± 2.97 ^aCP^	116.63 ± 5.42 ^cCP^	91.70 ± 4.03 ^eCP^	101.60 ± 3.20 ^dCP^	67.14 ± 6.10 ^fBP^	69.78 ± 4.26 ^fBP^
	Winter	137.33 ± 4.44 ^bAP^	152.50 ± 2.56 ^aBP^	128.92 ± 6.30 ^cBP^	98.53 ± 60 ^eBP^	106.82 ± 3.04 ^dBP^	73.60 ± 4.44 ^fAP^	76.58 ± 3.82 ^fAP^
	Spring	139.45 ± 3.72 ^bAP^	156.91 ± 3.54 ^aAP^	132.04 ± 2.35 ^cAP^	103.79 ± 5.43 ^eAP^	112.47 ± 3.15 ^dAP^	77.65 ± 3.97 ^fAP^	79.43 ± 3.52 ^fAP^
	**Mean**	**134.38 ± 6.84 ^X^**	**152.31 ± 4.89 ^X^**	**125.86 ± 8.30 ^X^**	**98.01 ± 7.11 ^X^**	**106.96 ± 5.43 ^X^**	**72.80 ± 6.46 ^X^**	**75.26 ± 5.56 ^X^**

The different letters (a, b, c, d, e, and f ) show the statistical results of “Duncan Multiple Range Test” (*p* < 0.05) among the fish tissues for the same site and season in the same row; The different letters (A, B and C) show the statistical results of “Duncan Multiple Range Test” (*p* < 0.05) among the seasons for the same site and fish tissues in the same column; The different letters (P and Q,) show the statistical results of “Duncan Multiple Range Test” (*p* < 0.05) among the sites for the same seasons and fish tissues in the same column; and the different letters (X) show the statistical results of “Duncan Multiple Range Test” (*p* < 0.05) among the means of sites for the same fish tissues in the same column.

**Table 4:** As concentration (mg kg^−1^) in tissue samples (Means ± SD) of *C. mrigala* caught from different sites at Panjnad headwork, Pakistan.

Sites	Seasons	Fish Tissues						
		Liver	Kidney	Gills	Fins	Skin	Muscles	Bones
**S1**	Autumn	17.04 ± 2.02 ^bCP^	21.09 ± 2.52 ^aCP^	12.42 ± 1.43 ^cCP^	11.77 ± 2.08 ^cdCP^	10.04 ± 1.98 ^dCP^	10.07 ± 1.96 ^dCP^	10.04 ± 1.70 ^dCP^
	Winter	23.29 ± 2.37 ^bBP^	26.77 ± 2.27 ^aBP^	19.71 ± 2.09 ^cBP^	16.73 ± 1.80 ^dBP^	16.07 ± 2.38 ^dBP^	13.89 ± 2.38 ^eBP^	15.29 ± 1.83 ^deBP^
	Spring	27.97 ± 2.39 ^bAP^	32.71 ± 2.43 ^aAP^	25.33 ± 2.10 ^cAP^	22.14 ± 2.32 ^dAP^	22.02 ± 2.49 ^dAP^	17.34 ± 1.09 ^eAP^	19.03 ± 2.21 ^eAP^
	**Mean**	**22.76 ± 5.05 ^X^**	**26.86 ± 5.36 ^X^**	**19.15 ± 5.69 ^X^**	**16.88 ± 4.75 ^X^**	**16.04 ± 5.45 ^X^**	**13.77 ± 3.53 ^X^**	**14.79 ± 4.19 ^X^**
**S2**	Autumn	17.28 ± 1.49 ^bCP^	21.45 ± 0.90 ^aCP^	11.56 ± 2.22 ^cCP^	11.35 ± 1.67 ^cCP^	10.01 ± 1.61 ^cdCP^	10.23 ± 0.98 ^cdCP^	9.59 ± 2.40 ^dCP^
	Winter	23.79 ± 2.14 ^bBP^	26.79 ± 1.88 ^aBP^	20.18 ± 1.98 ^cBP^	17.44 ± 2.12 ^dBP^	15.73 ± 1.17 ^deBP^	13.81 ± 2.14 ^fBP^	14.28 ± 1.79 ^efBP^
	Spring	27.93 ± 2.11 ^bAP^	31.87 ± 1.81 ^aAP^	23.05 ± 3.57 ^cAP^	20.10 ± 2.7 ^dAP^	20.16 ± 3.16 ^dAP^	15.71 ± 1.78 ^eAP^	18.72 ± 2.63 ^dAP^
	**Mean**	**23.00 ± 4.84 ^X^**	**26.70 ± 4.60 ^X^**	**18.26 ± 5.61 ^X^**	**16.30 ± 4.29 ^X^**	**15.30 ± 4.71 ^X^**	**13.25 ± 2.83 ^X^**	**14.20 ± 4.40 ^X^**
**S3**	Autumn	17.87 ± 1.68 ^bCP^	21.54 ± 2.55 ^aCP^	11.67 ± 2.44 ^cCP^	11.05 ± 1.27 ^cdCP^	10.96 ± 2.23 ^cdCP^	9.73 ± 1.84 ^cdCP^	9.24 ± 2.52 ^dCP^
	Winter	23.27 ± 2.87 ^bBP^	26.57 ± 2.45 ^aBP^	20.66 ± 1.56 ^cBP^	16.58 ± 2.87 ^dBP^	15.28 ± 1.92 ^dBP^	14.31 ± 2.65 ^dBP^	14.82 ± 2.46 ^dBP^
	Spring	28.03 ± 2.03 ^bAP^	32.08 ± 1.42 ^aAP^	24.73 ± 1.38 ^cAP^	20.47 ± 1.85 ^deAP^	20.85 ± 1.98 ^dAP^	16.86 ± 2.52 ^fAP^	18.73 ± 1.85 ^eAP^
	**Mean**	**23.05 ± 4.75 ^X^**	**26.73 ± 4.87 ^X^**	**19.02 ± 5.84 ^X^**	**16.03 ± 4.43 ^X^**	**15.70 ± 4.57 ^X^**	**13.63 ± 3.77 ^X^**	**14.26 ± 4.54 ^X^**

The different letters (a, b, c, d, e, and f) show the statistical results of “Duncan Multiple Range Test” (*p* < 0.05) among the fish tissues for the same site and season in the same row; The different letters (A, B, and C) show the statistical results of “Duncan Multiple Range Test” (*p* < 0.05) among the seasons for the same site and fish tissues in the same column; The different letters (P) show the statistical results of “Duncan Multiple Range Test” (*p* < 0.05) among the sites for the same seasons and fish tissues in the same column; and the different letters (X) show the statistical results of “Duncan Multiple Range Test” (*p* < 0.05) among the means of sites for the same fish tissues in the same column.

**Table 5:** Ba concentration (mg kg^−1^) in tissue samples (Means ± SD) of *C. mrigala* caught from different sites at Panjnad headwork, Pakistan.

Sites	Seasons	Fish Tissues						
		Liver	Kidney	Gills	Fins	Skin	Muscles	Bones
**S1**	Autumn	3.28 ± 0.61 ^aCP^	2.73 ± 0.59 ^aCP^	2.01 ± 0.67 ^bCP^	1.94 ± 0.51 ^bCP^	1.64 ± 0.62 ^bCP^	1.39 ± 0.70 ^bCP^	1.38 ± 0.83 ^bCP^
	Winter	4.65 ± 0.76 ^aBP^	4.76 ± 0.75 ^aBP^	4.32 ± 0.63 ^abBP^	3.80 ± 0.77 ^bcBP^	3.69 ± 0.98 ^bcBP^	3.29 ± 0.73 ^cdBP^	2.89 ± 0.92 ^dBP^
	Spring	7.43 ± 0.70 ^aAP^	7.11 ± 0.68 ^aAP^	7.03 ± 0.47 ^aAP^	6.26 ± 0.52 ^bAP^	6.15 ± 0.63 ^bAP^	5.80 ± 0.40 ^bAP^	5.69 ± 0.96 ^bAP^
	**Mean**	**5.12 ± 1.88 ^X^**	**4.86 ± 1.93 ^X^**	**4.45 ± 2.17 ^X^**	**4.00 ± 1.90 ^X^**	**3.83 ± 2.01 ^X^**	**3.49 ± 1.94 ^X^**	**3.32 ± 2.01 ^X^**
**S2**	Autumn	2.75 ± 0.52 ^aCQ^	2.55 ± 0.28 ^aCP^	2.00 ± 0.63 ^bCP^	1.54 ± 0.29 ^cCPQ^	1.33 ± 0.30 ^cCP^	1.43 ± 0.54 ^cCP^	1.25 ± 0.42 ^cCP^
	Winter	4.62 ± 0.32 ^aBP^	4.00 ± 0.36 ^bBQ^	3.89 ± 0.51 ^bBP^	3.18 ± 0.62 ^cBP^	3.16 ± 0.49 ^cBP^	2.93 ± 0.38 ^cBP^	3.08 ± 0.31 ^cBP^
	Spring	5.63 ± 0.69 ^aAR^	5.05 ± 0.48 ^bAR^	5.15 ± 0.62 ^abAR^	4.65 ± 0.42 ^bAR^	4.11 ± 0.40 ^cAR^	3.74 ± 0.63 ^cAR^	3.85 ± 0.31 ^cAR^
	**Mean**	**4.34 ± 1.32 ^X^**	**3.87 ± 1.11 ^Y^**	**3.68 ± 1.43 ^X^**	**3.12 ± 1.37 ^X^**	**2.87 ± 1.24 ^Y^**	**2.70 ± 1.10 ^X^**	**2.73 ± 1.16 ^X^**
**S3**	Autumn	2.59 ± 0.35 ^aCQ^	2.59 ± 0.42 ^aCP^	2.33 ± 0.28 ^aCP^	1.47 ± 0.37 ^bCQ^	1.45 ± 0.51 ^bCP^	1.19 ± 0.31 ^bCP^	1.23 ± 0.36 ^bCP^
	Winter	4.66 ± 0.59 ^aBP^	4.28 ± 0.89 ^abcBPQ^	4.38 ± 0.81 ^abBP^	3.77 ± 0.70 ^bc dBP^	3.54 ± 0.73 ^cdBP^	3.40 ± 0.82 ^dBP^	3.55 ± 0.73 ^cdBP^
	Spring	6.24 ± 0.43 ^aAQ^	5.97 ± 0.40 ^aAQ^	5.92 ± 0.55 ^aAQ^	5.37 ± 0.69 ^bAQ^	5.06 ± 0.40 ^bcAQ^	4.70 ± 0.53 ^cdAQ^	4.50 ± 0.26 ^dAQ^
	**Mean**	**4.50 ± 1.59 ^X^**	**4.28 ± 1.53 ^XY^**	**4.21 ± 1.60 ^X^**	**3.54 ± 1.73 ^X^**	**3.35 ± 1.60 ^XY^**	**3.10 ± 1.58 ^X^**	**3.09 ± 1.48 ^X^**

The different letters (a, b, c, and d show the statistical results of “Duncan Multiple Range Test” (*p* < 0.05) among the fish tissues for the same site and season in the row line; The different letters (A, B and C) show the statistical results of “Duncan Multiple Range Test” (*p* < 0.05) among the seasons for the same site and fish tissues in the same column; The different letters (P, Q and R) show the statistical results of “Duncan Multiple Range Test” (*p* < 0.05) among the sites for the same seasons and fish tissues in the same column; and the different letters (X and Y) show the statistical results of “Duncan Multiple Range Test” (*p* < 0.05) among the means of sites for the same fish tissues in the same column.

**Table 6:** Pb concentration (mg kg^−1^) in tissue samples (Means ± SD) of *C. mrigala* caught from different sites at Panjnad headwork, Pakistan.

Sites	Seasons	Fish Tissues						
		Liver	Kidney	Gills	Fins	Skin	Muscles	Bones
**SI**	Autumn	2.79 ± 0.51 ^aCP^	2.37 ± 0.63 ^abCP^	2.23 ± 0.42 ^bcCP^	1.73 ± 0.45 ^cdCP^	1.55 ± 0.63 ^dCP^	1.22 ± 0.58 ^dCP^	1.34 ± 0.55 ^dCP^
	Winter	4.36 ± 1.06 ^aBP^	4.10 ± 1.16 ^aBP^	3.48 ± 1.08 ^abBP^	2.80 ± 0.47 ^bcBQ^	2.79 ± 0.91 ^bcBP^	2.43 ± 1.01 ^cBP^	2.26 ± 1.00 ^cBP^
	Spring	7.05 ± 0.87 ^abAP^	7.57 ± 0.42 ^aAP^	6.60 ± 0.65 ^bAP^	4.78 ± 0.38 ^cAP^	4.24 ± 0.42 ^dAQ^	4.22 ± 0.40 ^dAPQ^	3.99 ± 0.63 ^dAP^
	**Mean**	**4.73 ± 1.96 ^X^**	**4.68 ± 2.34 ^X^**	**4.10 ± 2.01 ^X^**	**3.10 ± 1.36 ^X^**	**2.86 ± 1.30 ^X^**	**2.62 ± 1.43 ^X^**	**2.53 ± 1.33 ^X^**
**SII**	Autumn	2.44 ± 0.36 ^aCP^	2.25 ± 0.37 ^aCP^	1.82 ± 0.33 ^bCP^	1.46 ± 0.46 ^cCP^	1.24 ± 0.32 ^cCP^	1.12 ± 0.34 ^cCP^	0.77 ± 0.32 ^dCQ^
	Winter	4.24 ± 0.44 ^aBP^	4.03 ± 0.49 ^abBP^	3.56 ± 0.44 ^bcBP^	3.41 ± 0.67 ^cBPQ^	3.28 ± 0.54 ^cBP^	2.65 ± 0.66 ^dBP^	2.51 ± 0.63 ^dBP^
	Spring	6.32 ± 0.71 ^aAP^	6.45 ± 0.95 ^aAQ^	5.45 ± 1.16 ^bAQ^	5.00 ± 0.50 ^bAP^	4.85 ± 0.64 ^bAP^	3.94 ± 0.36 ^cAQ^	4.02 ± 0.37 ^cAP^
	**Mean**	**4.34 ± 1.69 ^X^**	**4.24 ± 1.86 ^X^**	**3.61 ± 1.67 ^X^**	**3.29 ± 1.57 ^X^**	**3.12 ± 1.59 ^X^**	**2.57 ± 1.26 ^X^**	**2.43 ± 1.42 ^X^**
**SIII**	Autumn	2.83 ± 0.38 ^aCP^	2.77 ± 0.61 ^aCP^	1.83 ± 0.81 ^bCP^	1.39 ± 0.73 ^bcCP^	1.12 ± 0.56 ^cCP^	1.05 ± 0.60 ^cCP^	0.80 ± 0.59 ^cCQ^
	Winter	4.36 ± 1.08 ^aBP^	4.10 ± 0.80 ^abBP^	4.03 ± 1.20 ^abBP^	3.64 ± 0.91 ^abcBP^	3.30 ± 0.83 ^bcBP^	2.95 ± 1.06 ^cBP^	2.84 ± 1.01 ^cBP^
	Spring	6.82 ± 0.76 ^aAP^	6.68 ± 0.65 ^aAQ^	6.04 ± 0.65 ^bAPQ^	4.89 ± 0.51 ^cAP^	4.91 ± 0.57 ^cAP^	4.50 ± 0.37 ^cdAP^	4.26 ± 0.55 ^dAP^
	**Mean**	**4.67 ± 1.84 ^X^**	**4.52 ± 1.78 ^X^**	**3.97 ± 1.96 ^X^**	**3.30 ± 1.64 ^X^**	**3.11 ± 1.71 ^X^**	**2.83 ± 1.60 ^X^**	**2.63 ± 1.62 ^X^**

The different letters (a, b, c, and d) show the statistical results of “Duncan Multiple Range Test” (*p* < 0.05) among the fish tissues for the same site and season in the row line; The different letters (A, B, and C) show the statistical results of “Duncan Multiple Range Test” (*p* < 0.05) among the seasons for the same site and fish tissues in the same column; The different letters (P and Q) show the statistical results of “Duncan Multiple Range Test” (*p* < 0.05) among the sites for the same seasons and fish tissues in the same column; and the different letters (X) show the statistical results of “Duncan Multiple Range Test” (*p* < 0.05) among the means of sites for the same fish tissues in the same column.

**Table 7:** Correlation among different sampling months (October 2021–May 2022) for Al accumulation in different organs of *C. mrigala* at Panjnad headwork. Bold values represent statistically significant values.

	September	October	November	December	January	February	March	April
**Liver**								
October	−0.275							
November	0.186	0.253						
December	−0.276	0.064	−0.321					
January	−0.023	**−0.819**	−0.085	−0.187				
February	0.206	0.054	0.290	−0.023	−0.068			
March	0.171	**−0.701**	−0.246	−0.198	0.512	0.411		
April	0.172	−0.149	0.371	−0.226	−0.035	−0.303	0.151	
May	−0.196	−0.313	−0.653	−0.219	0.441	−0.304	0.420	−0.173
**Kidney**								
October	−0.106							
November	−0.220	−0.376						
December	−0.372	0.377	0.394					
January	−0.450	−0.461	0.160	0.163				
February	0.373	0.175	−0.314	0.068	−0.247			
March	−0.352	−0.039	**0.680**	0.464	0.303	−0.354		
April	0.372	−0.071	−0.371	−0.559	0.048	0.393	−0.047	
May	−0.237	−0.127	0.039	0.403	0.068	0.210	0.092	−0.229
**Gills**								
October	0.028							
November	−0.055	0.146						
December	−0.126	−0.178	0.112					
January	−0.235	0.267	0.497	−0.172				
February	−0.304	0.620	−0.441	−0.350	−0.041			
March	−0.095	0.131	−0.091	−0.620	−0.417	0.373		
April	0.297	0.171	**0.685**	0.228	0.260	−0.299	−0.275	
May	**0.750**	−0.363	−0.104	0.121	−0.250	−0.670	−0.197	0.080
**Fins**								
October	−0.392							
November	0.328	−0.250						
December	0.198	−0.120	−0.494					
January	0.630	−0.358	−0.169	0.373				
February	0.108	−0.492	0.642	−0.499	−0.388			
March	−0.051	−0.425	0.049	0.614	0.084	−0.012		
April	**0.689**	−0.108	0.201	0.147	0.299	−0.037	−0.179	
May	−0.006	−0.214	−0.374	−0.211	0.480	−0.291	−0.366	−0.217
**Skin**								
October	−0.428							
November	0.213	**−0.732**						
December	0.442	0.388	−0.257					
January	−0.043	0.152	−0.544	−0.275				
February	0.127	−0.349	0.307	−0.022	−0.197			
March	−0.073	−0.467	0.302	−0.312	−0.465	0.142		
April	−0.020	−0.608	0.456	−0.225	0.070	0.601	0.082	
May	−0.274	0.089	−0.183	−0.017	−0.252	−0.663	0.362	−0.363
**Muscles**								
October	0.227							
November	−0.414	−0.564						
December	−0.052	−0.532	0.514					
January	0.119	0.085	0.028	0.092				
February	−0.499	−0.312	0.222	−0.105	**−0.857**			
March	−0.544	−0.395	0.861	0.335	0.032	0.151		
April	0.168	0.031	−0.256	0.317	**0.674**	−0.636	−0.387	
May	−0.323	0.294	0.435	−0.043	0.542	−0.364	0.532	−0.007
**Bones**								
October	−0.357							
November	0.546	0.170						
December	0.122	0.397	0.133					
January	0.064	−0.216	0.310	0.243				
February	**0.692**	−0.187	0.331	−0.059	0.272			
March	−0.309	0.404	0.417	0.180	0.302	−0.351		
April	0.307	**−0.676**	−0.242	−0.356	0.005	0.048	−0.408	
May	**0.817**	0.102	0.643	0.452	0.034	0.561	0.117	−0.116

**Table 8:** Correlation among different sampling months (October 2021–May 2022) for As accumulation in different organs of *C. mrigala* at Panjnad headwork. Bold values represent statistically significant values.

	September	October	November	December	January	February	March	April
**Liver**								
October	0.208							
November	0.160	−0.454						
December	−0.291	−0.135	−0.485					
January	0.408	−0.406	0.266	−0.017				
February	0.152	−0.051	0.402	−0.077	0.522			
March	**−0.717**	−0.290	−0.158	0.565	−0.254	0.024		
April	0.021	0.237	**−0.743**	0.326	0.101	−0.216	0.015	
May	−0.341	−0.306	−0.006	−0.298	0.373	−0.055	−0.074	0.349
**Kidney**								
October	0.267							
November	**−0.775**	−0.076						
December	**0.796**	−0.063	**−0.884**					
January	−0.017	−0.104	−0.173	0.405				
February	−0.302	0.580	0.235	−0.439	−0.449			
March	0.034	−0.414	0.054	0.067	0.321	−0.509		
April	−0.267	−0.150	0.127	−0.324	−0.344	0.025	−0.330	
May	−0.358	**−0.672**	0.265	0.052	0.442	−0.529	0.098	−0.148
**Gills**								
October	−0.047							
November	0.012	**−0.682**						
December	0.238	−0.287	0.471					
January	−0.109	**0.700**	−0.547	0.150				
February	−0.133	0.336	−0.060	0.088	0.095			
March	−0.251	−0.506	0.411	0.373	−0.363	−0.438		
April	0.233	0.025	−0.463	−0.298	−0.300	0.098	0.120	
May	0.564	−0.508	0.393	0.158	−0.340	0.012	−0.292	−0.102
**Fins**								
October	0.049							
November	0.298	0.148						
December	0.286	0.241	0.560					
January	−0.387	0.321	−0.160	−0.083				
February	0.319	−0.449	0.046	−0.136	−0.320			
March	0.038	−0.178	−0.148	−0.271	−0.440	−0.161		
April	**−0.756**	−0.325	−0.034	−0.202	−0.058	0.070	0.152	
May	0.246	0.042	0.491	**0.802**	−0.498	−0.176	0.309	0.019
**Skin**								
October	0.444							
November	0.416	**0.699**						
December	0.134	0.082	−0.071					
January	0.390	−0.021	0.077	−0.115				
February	0.096	0.161	0.002	−0.488	0.518			
March	−0.211	0.233	0.370	0.119	0.063	0.262		
April	−0.117	−0.422	−0.430	0.568	−0.258	−0.222	−0.164	
May	0.233	−0.218	0.141	**−0.773**	0.498	0.341	−0.100	−0.607
**Muscles**								
October	0.659							
November	−0.008	−0.300						
December	0.263	0.283	−0.118					
January	−0.031	−0.252	**0.907**	−0.126				
February	−0.264	−0.136	−0.222	−0.515	−0.510			
March	0.136	0.295	−0.284	0.474	−0.036	−0.509		
April	0.002	−0.357	0.074	−0.348	0.143	−0.014	0.167	
May	−0.440	−0.154	−0.196	−0.218	−0.289	0.582	0.139	0.372
**Bones**								
October	0.196							
November	−0.229	0.155						
December	0.057	0.156	**−0.673**					
January	−0.194	−0.563	0.167	−0.326				
February	−0.316	0.290	0.118	0.498	−0.012			
March	0.626	−0.267	−0.172	−0.011	−0.108	−0.257		
April	−0.167	−0.520	0.136	−0.652	0.417	**−0.726**	0.162	
May	0.002	0.589	−0.419	0.371	−0.363	0.395	−0.226	−0.510

**Table 9:** Correlation among different sampling months (October 2021–May 2022) for Ba accumulation in different organs of *C. mrigala* at Panjnad headwork. Bold values represent statistically significant values.

	September	October	November	December	January	February	March	April
**Liver**								
October	0.411							
November	0.299	0.637						
December	−0.213	−0.043	−0.358					
January	−0.116	−0.115	−0.492	−0.191				
February	0.637	**0.670**	**0.791**	−0.443	−0.082			
March	0.594	0.551	0.632	**−0.731**	−0.029	**0.811**		
April	0.570	0.412	0.604	**−0.721**	0.041	**0.877**	**0.934**	
May	0.169	0.502	**0.754**	**−0.685**	−0.156	0.667	**0.840**	**0.745**
**Kidney**								
October	0.413							
November	−0.538	−0.017						
December	0.017	−0.031	0.181					
January	**−0.711**	−0.031	0.471	−0.625				
February	−0.340	0.197	0.433	−0.239	0.619			
March	−0.179	0.448	0.395	−0.326	0.598	**0.925**		
April	−0.360	0.405	0.448	0.166	0.412	**0.836**	**0.824**	
May	−0.339	0.229	0.662	−0.034	0.511	**0.872**	**0.892**	**0.818**
**Gills**								
October	**0.771**							
November	−0.617	**−0.775**						
December	−0.408	**−0.746**	**0.745**					
January	**0.717**	0.616	−0.618	−0.465				
February	0.315	0.497	−0.421	**−0.835**	0.350			
March	0.048	0.179	−0.034	−0.560	0.311	**0.862**		
April	−0.328	−0.208	0.271	−0.194	0.045	0.597	**0.864**	
May	−0.233	−0.015	0.182	−0.465	0.009	**0.737**	**0.924**	**0.840**
**Fins**								
October	−0.503							
November	−0.163	0.528						
December	**−0.693**	0.178	−0.431					
January	0.373	−0.079	0.081	−0.521				
February	−0.125	0.238	**0.816**	−0.484	0.522			
March	−0.335	0.593	**0.671**	−0.322	0.477	**0.779**		
April	−0.533	0.624	0.633	−0.115	0.259	**0.707**	**0.915**	
May	−0.278	0.399	0.474	−0.318	0.631	**0.721**	**0.912**	**0.756**
**Skin**								
October	0.006							
November	−0.119	0.349						
December	0.340	−0.037	−0.659					
January	0.034	−0.066	**0.867**	−0.660				
February	−0.205	0.282	**0.979**	−0.617	**0.879**			
March	−0.152	0.273	**0.950**	−0.566	**0.890**	**0.984**		
April	−0.308	0.423	**0.883**	−0.388	0.617	**0.896**	**0.837**	
May	−0.385	0.235	**0.819**	−0.331	0.635	**0.871**	**0.813**	**0.962**
**Muscles**								
October	0.363							
November	−0.187	0.242						
December	0.472	0.511	0.290					
January	0.100	−0.334	0.076	−0.515				
February	−0.269	−0.417	−0.093	−0.587	0.579			
March	−0.595	−0.407	0.153	−0.469	0.262	**0.768**		
April	−0.470	−0.017	0.392	−0.149	0.174	**0.686**	**0.862**	
May	−0.092	0.190	0.196	−0.045	0.173	**0.693**	0.614	**0.867**
**Bones**								
October	−0.502							
November	−0.338	0.122						
December	0.063	**0.683**	−0.357					
January	0.296	−0.134	−0.631	0.075				
February	0.066	−0.642	0.034	**−0.710**	0.507			
March	−0.205	−0.398	0.448	−0.666	0.170	**0.787**		
April	−0.470	−0.109	**0.858**	**−0.691**	−0.347	0.464	**0.749**	
May	−0.238	−0.447	**0.736**	**−0.790**	−0.477	0.429	**0.735**	**0.860**

**Table 10:** Correlation among different sampling months (October 2021–May 2022) for Pb accumulation in different organs of *C. mrigala* at Panjnad headwork. Bold values represent statistically significant values.

	September	October	November	December	January	February	March	April
**Liver**								
October	−0.271							
November	0.165	0.511						
December	−0.413	−0.433	**−0.732**					
January	0.541	−0.084	0.261	−0.586				
February	0.098	0.347	**0.818**	−0.530	−0.151			
March	−0.181	0.134	0.637	−0.377	0.131	**0.683**		
April	0.143	0.405	**0.759**	**−0.679**	0.311	**0.749**	**0.839**	
May	0.041	**0.725**	0.547	−0.431	−0.111	0.450	0.242	0.432
**Kidney**								
October	0.468							
November	0.256	0.568						
December	0.019	−0.033	−0.240					
January	0.044	**0.747**	0.651	−0.181				
February	−0.407	−0.411	0.369	−0.574	−0.095			
March	−0.274	−0.299	0.442	−0.639	−0.021	**0.956**		
April	−0.273	−0.488	0.034	−0.601	−0.258	**0.684**	0.544	
May	−0.270	−0.616	0.057	−0.473	−0.338	**0.830**	**0.723**	**0.697**
**Gills**								
October	−0.437							
November	0.190	0.349						
December	0.216	−0.239	−0.640					
January	−0.938	0.571	0.016	−0.204				
February	−0.366	0.458	**0.735**	**−0.892**	0.421			
March	0.018	0.405	**0.811**	**−0.733**	0.137	**0.830**		
April	0.255	−0.024	**0.852**	−0.536	−0.119	0.569	0.492	
May	0.297	−0.477	0.140	−0.165	−0.291	0.098	0.411	0.011
**Fins**								
October	−0.297							
November	0.111	−0.333						
December	0.584	−0.259	−0.342					
January	**−0.825**	0.015	−0.002	−0.289				
February	−0.578	−0.074	−0.064	0.159	**0.811**			
March	−0.343	0.436	−0.480	0.068	0.047	0.248		
April	0.302	−0.177	0.078	0.370	−0.277	−0.109	−0.338	
May	−0.565	0.118	−0.206	−0.126	0.260	0.359	0.466	0.415
**Skin**								
October	−0.490							
November	0.461	0.188						
December	0.455	−0.266	−0.213					
January	**−0.686**	0.527	−0.596	−0.119				
February	0.199	−0.276	0.137	−0.457	−0.155			
March	−0.526	0.546	−0.133	−0.249	**0.806**	0.003		
April	0.194	−0.309	−0.481	0.859	0.174	−0.175	0.027	
May	−0.540	0.550	−0.506	0.247	**0.862**	−0.404	**0.672**	0.420
**Muscles**								
October	−0.093							
November	−0.419	0.170						
December	**0.794**	−0.177	−0.512					
January	−0.270	−0.044	−0.288	−0.162				
February	−0.444	−0.086	0.635	**−0.698**	0.028			
March	−0.591	0.106	0.501	−0.477	**0.672**	0.447		
April	−0.087	0.617	−0.117	−0.233	0.619	0.190	0.436	
May	−0.529	0.491	0.599	**−0.825**	0.145	0.630	0.521	0.480
**Bones**								
October	−0.007							
November	0.287	**0.685**						
December	0.205	**−0.819**	**−0.700**					
January	**−0.726**	0.028	−0.219	−0.370				
February	**−0.723**	0.476	0.169	−0.649	0.562			
March	0.030	−0.481	−0.460	0.282	−0.006	−0.179		
April	−0.172	−0.312	−0.476	0.159	0.469	0.029	0.515	
May	0.146	0.318	0.302	−0.545	0.300	0.232	−0.302	−0.266

**Table 11:** Human Health Risk Assessment of heavy metals in *C. mrigala*.

Metal	*^†^Ing_Ex_*	*THQ_Ing_*	*TTHQ_Ing_*	*CR_Ing_*
Al	23.05	0.02	3.76	-
As	4.3	0.02		6.4742
Ba	0.98	3.27		-
Pb	0.89	0.44		0.0076

*^†^Ing_Ex_*= Ingestion exposure; *THQ_Ing_* = Target hazard quotient via ingestion; *TTHQ_Ing_* = Total target hazard quotient through oral intake; *CR_Ing_* = Carcinogenic risk via oral ingestion.

## Data Availability

Data is contained within the article.
